# Relationship between Volatile Organic Compounds and Microorganisms Isolated from Raw Sheep Milk Cheeses Determined by Sanger Sequencing and GC–IMS

**DOI:** 10.3390/foods12020372

**Published:** 2023-01-13

**Authors:** María J. Ruiz, José A. Salatti-Dorado, María J. Cardador, Laureano Frizzo, Rafael Jordano, Lourdes Arce, Luis M. Medina

**Affiliations:** 1 Laboratory of Food Analysis “Rodolfo Oscar Dalla Santina”, Institute of Veterinary Science (ICiVet Litoral), National University of the Litoral-National Council of Scientific and Technical Research (UNL/CONICET), Esperanza 3080, Province of Santa Fe, Argentina; 2 Analytical Chemistry Department, Institute of Fine Chemistry and Nanochemistry, International Agrifood Campus of Excellence, Marie Curie Annex Building, Campus de Rabanales, University of Cordoba, E-14071 Cordoba, Spain; 3 Food Science and Technology Department, International Agrifood Campus of Excellence, Charles Darwin Annex Building, Campus de Rabanales, University of Córdoba, E-14071 Cordoba, Spain

**Keywords:** lactic acid bacteria, GC–IMS, artisan cheese, volatile organic compounds, VOC

## Abstract

Recently, the interest of consumers regarding artisan cheeses worldwide has increased. The ability of different autochthonous and characterized lactic acid bacteria (LAB) to produce aromas and the identification of the volatile organic compounds (VOCs) responsible for flavor in cheeses are important aspects to consider when selecting strains with optimal aromatic properties, resulting in the diversification of cheese products. The objective of this work is to determine the relationship between VOCs and microorganisms isolated (*Lacticaseibacillus paracasei*, *Lactiplantibacillus plantarum*, *Leuconostoc mesenteroides* and *Lactococcus lactis* subsp. *hordniae*) from raw sheep milk cheeses (matured and creamy natural) using accuracy and alternative methods. On combining Sanger sequencing for LAB identification with Gas Chromatography coupled to Ion Mobility Spectrometry (GC–IMS) to determinate VOCs, we describe cheeses and differentiate the potential role of each microorganism in their volatilome. The contribution of each LAB can be described according to their different VOC profile. Differences between LAB behavior in each cheese are shown, especially between LAB involved in creamy cheeses. Only L. *lactis* subsp. *hordniae* and L. *mesenteroides* show the same VOC profile in de Man Rogosa and Sharpe (MRS) cultures, but for different cheeses, and show two differences in VOC production in skim milk cultures. The occurrence of *Lactococcus lactis* subsp. *hordniae* from cheese is reported for first time.

## 1. Introduction

The progressive consumer awareness of healthy, low (or no-)-processed and sustainable products is a fact, with artisanal products (including raw milk cheeses) being preferred in many cases [1]. In recent years, the interest of consumers regarding artisan cheeses worldwide increased [2]. Based on regulations focused on food safety and nutrition and the consumer trend towards products that improve welfare, functional dairy products have become a leading sector in the food industry. Sheep’s milk is considered a functional food due to its nutritional quality [3]. Autochthonous microorganisms metabolize the raw materials during fermentation. This process enriches the nutritional value in some fermented foods, imparts health benefits to the consumers and generates gastronomic diversity [4]. In fact, starter and nonstarter lactic acid bacteria (LAB) and coagulating enzymes, used for the production and ripening of cheese, are able to transform protein, fat, lactose and citrate into volatile compounds, enhancing the formation of essential aroma compounds [5]. Raw milk contains natural enzymes, and its microbiota generates and enhances characteristic flavors appreciated by consumers through their metabolic activity. Autochthonous lactic acid bacteria (LAB) are a group of microorganisms including many of those commonly considered as probiotics, as they exert a beneficial effect on the gastrointestinal tract of humans and animals. Furthermore, many LAB genera, such as *Lactobacillus*, *Lactococcus*, *Leuconostoc* and the recently classified *Lactiplantibacillus* and *Lacticaseibacillus* are responsible for the organoleptic characteristics of many traditional foods, improving the nutritional value in some fermented foods [6]. Fermentation processes and food processing and preservation techniques are responsible for the organoleptic characteristics, such as flavor and texture, of this type of food. In this sense, the relationship between volatile organic compounds (VOCs) emitted from raw milk cheeses and the starter and non-starter LAB involved is a matter of recent interest [7].

In the last decade, legislation and market strategies to control and standardize the food supply chain have motivated the development of tools for the authentication of food products [8]. A simple, fast and sensitive analytical technique that has been found in recent years to establish aromatic fingerprints is gas chromatography in conjunction with ion mobility spectrometry (GC–IMS) [9] by extracting VOCs that are generated in the headspace (HS) of the vial. This technique makes it possible to detect VOCs at a part-per-billion (ppb) level in real time in many liquid and solid samples and without any prior treatment. The sensitivity and selectivity of the IMS make it a very suitable tool for use in quality control and food safety, as well as in the characterization, authentication and traceability of food [10]. In fact, GC–IMS can currently be considered an excellent method to separate and identify the volatile contributors to food flavor. It has been already employed to determine the VOC profiles of milk, cheeses and other dairy products [11,12,13].

Regarding the use of different LAB and their greater or lesser probiotic potential, the conditions are specific for each strain [14]. For this reason, genetic identification is essential for the characterization of this type of bacteria. Molecular analysis based on 16S rRNA, like Sanger sequencing, is one of the methods currently used based on its precision and costs, obtaining bacterial genetic profiles [15].

The objective of this work is to determine the relationship between VOCs and microorganisms isolated from raw sheep milk cheeses, using accuracy and alternative methods. In this way, we identified LAB isolated from artisanal raw sheep milk cheeses using Sanger sequencing and VOCs were detected by GC–IMS.

## 2. Materials and Methods

### 2.1. Origin, Isolation and Preparation of Samples and Bacterial Cultures

Six samples of cheese made from raw sheep milk were analyzed. Three samples corresponded to a creamy natural cheese called “Torta del Casar” from Extremadura, Spain. The other three samples were extracted from a matured cheese from Navarra, Spain. In both cheeses, the samples were taken from different parts of the cheese (center, inner and rind areas). A total of 10 g of each sample was weighed, suspended in 90 mL of 0.1% peptone water and homogenized in a Stomacher^®^ Lab Blender (Seward, UK) for 120 s at 230 rpm. Then, 0.1 mL of the 1:10^7^ dilution was spread on Man Rogosa and Sharpe agar (MRS, Oxoid, UK) in duplicate. Half of the plates were incubated in anaerobic jars (Thermo Scientific™, Oxoid, UK) at 37 °C for 48 h; the other half were incubated in aerobic conditions at 30 °C for 72 h. Presumptive colonies of LAB were cultured in 3 mL of MRS broth (Oxoid, UK) and incubated in an anaerobic jar (Thermo Scientific™, Oxoid, UK) at 37 °C for 24 h and in aerobiosis at 30 °C for 48 h. Growth colonies were examined for Gram, morphology and catalase determinations prior to bacterial identification by Sanger sequencing.

For the evaluation of LAB spectral fingerprints by GC–IMS, the identified microorganisms were activated in MRS broth (Oxoid, UK) for 24 h at 37 °C, and cultured (pour-plating) on the same agarized medium. Finally, a colony from each culture was collected and suspended in 5 mL of MRS broth (Oxoid, UK) and incubated under the same conditions. Cell concentration was measured by optical density at 540 nm (OD_540_) in a spectrophotometer (ThermoFisher, Waltham, MA, USA).

### 2.2. Bacterial Identification by Sanger Sequencing

Sanger sequencing was used as a genotypic characterization method, standardized by the Central Research Support Service of the University of Córdoba, Spain. DNA was extracted from 1.5 mL of culture using the Higher Purity Bacterial Genomic DNA Isolation kit (Canvax, Valladolid, Spain). Good-quality DNA with a ratio of 260/280 between 1.8–2.0 was obtained. A total of 25 ng of DNA was taken to PCR amplify the 16S ribosomal DNA with the universal primers 16SFw: 5’-AGAGTTTGATCCTGGCTCAG-3’ and 16SRv: 5’-GAAAGGAGGTGATCCAGCCG-3’. The reaction was carried out in a 20 µL solution containing 1x reaction buffer, 2.5 mM MgCl_2_, 100 µM of each dNTP, 0.25 µM of each primer, 1% DMSO and 1 u of BioTaq Taq Polymerase (BioTools). The amplification program includes a first denaturation step at 95 °C for 5 min, followed by 30 cycles of 1 min at 95 °C, 1 min at 55 °C and 1 min at 72 °C, and a final extension step of 7 min at 72 °C. The PCR reactions were purified by precipitation with AcNa/EtOH and suspended in 15 µL of Milli-Q ultrapure water. For each sequencing reaction (16SFw and 16SRv), 6 µL of purified DNA was taken. The sequences obtained from each isolate were aligned to achieve the complete 16S ribosomal sequence (DNAstar megalign program) and analyzed by BLAST in the GeneBank database (https://blast.ncbi.nlm.nih.gov, access on 5 September 2021).

### 2.3. GC–IMS Analysis Conditions

The analyses of the VOC profiles were made on a headspace gas chromatography-ion mobility spectrometer (HS-GC–IMS) instrument (FlavourSpec^®^, G.A.S. Gesellschaft für analytische Sensorsysteme mbH. Dortmund, Germany). The accuracy of the GC–IMS method was checked using ketone mix standards. In addition, empty sterile glass vials with magnetic screw caps and septums were analyzed as blanks to determine the presence of compounds retained on the GC column and in the IMS drift tube and run, if necessary, a pre-cleaning method. Working conditions were used according to Gallegos et al. [4] with modifications. Vials were heated at 70 °C for 5 min with a stirring speed of 500 rpm. The injector temperature was 80 °C. A 500 µL aliquot of the headspace was injected into the heated injector and then transferred to a non-polar GC column (SE-54-CB from CS-Chromatographie Service GmbH, Düren, Germany), with a stationary phase formed by 94% methyl–5% phenyl–1% vinyl silicone, with a film thickness of 0.25 µm, 30 m length and 0.25 mm internal diameter. The GC column was operated at a constant temperature (40 °C). The flow rate of N_2_ used as sample gas was 5 mL min^−1^. N_2_ was also used as a drift gas at a constant flow of 250 mL min^−1^. The drift gas enters the device in the opposite direction to the ions, to prevent non-ionized molecules or analytes from entering the ionization chamber. The N_2_ of purity grade 5.0 was supplied by Al Air Liquid Spain S.A. (Madrid, Spain). The ions are formed in the tritium source ionization chamber and focused on a louvered grating (BN gate, grating pulse width = 100 µs), and begin to move along the length of the 10 cm long drift tube maintained at 65 °C, under a constant electric field of 400 V cm^−1^. The separated ions arrive at the detector consisting of a Faraday plate. Data were acquired and recorded during 30 min of analysis. The spectrometer was driven in the positive drift voltage mode. The study of the VOCs of the reference analytes, LAB cultures and cheeses and the analysis of blanks between each sample were carried out. In this way, contamination and non-real signals are avoided.

### 2.4. Identification of VOCs

Chemical standards were used to unequivocally confirm the presence of volatile compounds in LAB cultures and cheese samples. The identification of volatile metabolites was carried out by analysis of individual standards at 1 or 2 mg L^−1^ by GC–IMS. The commercial software VOCal version 0.2.9 (G.A.S. Gesellschaft für analytische Sensorsysteme mbH. Dortmund, Germany) was used to acquire the data. The coordinate positions (drift time and retention time) of a particular signal were compared to the database to identify the associated compound or metabolite. Identification was performed through comparison of retention and drift time in samples with those of the individual standards and confirmed using a commercial GC–IMS library (Library Plot Module Version 0.1., G.A.S. Gesellschaft für analytische Sensorsysteme mbH, Dortmund, Germany).

The different analytical-grade VOCs (Sigma-Aldrich, St. Louis, MO, USA) were chosen according to studies based on the detection of VOCs in LAB samples and in cheese samples made from raw sheep milk. The compounds studied in this work are summarized in Table 1. Stock solutions at 1000 mg L^−1^ for each compound were prepared by dissolving the appropriate volume of each compound in Milli-Q ultrapure water. Working solutions at 1 mg L^−1^ were prepared by dilution of stock solutions with Milli-Q water. All solutions were stored away from light at 4 °C. The MRS broth (culture medium) was also analyzed to identify the signals corresponding to this solution.

### 2.5. Analysis of Samples

LAB cultures were analyzed with a previous growth of 24 h at 37 °C. Each bacterial culture (1 mL) was poured into a 20 mL glass vial closed with a magnetic screw cap and septum. The vials were individually subjected to GC–IMS analysis using the parameters already mentioned. 

### 2.6. Data Analysis

GC–IMS data analysis was performed with VOCal software version 0.2.9 and VOCal’s extended functions: Reporter Plot Version 0.1. and Gallery Plot Module Version 0.1. (G.A.S. Gesellschaft für analytische Sensorsysteme mbH. Dortmund, Germany). A comparative analysis between VOC results was performed using Fisher’s exact test.

## 3. Results and Discussion

### 3.1. Sequencing of LAB Isolated from Raw Sheep Milk Cheeses

The four LAB strains isolated from matured cheese and creamy natural cheese made with raw sheep milk were identified by Sanger sequencing as *Lacticaseibacillus paracasei*, *Lactiplantibacillus plantarum* (formerly included in *Lactobacillus* spp.), *Lactococcus lactis* subsp. *hordniae* and *Leuconostoc mesenteroides*. *L. paracasei* and *L. lactis* subsp. *hordniae* strains were isolated from matured cheese and *L. plantarum* and *L. mesenteroides* strains were isolated from natural creamy cheese. Genomic identification showed 99% homology with the NCBI database. To the best of the authors’ knowledge, the occurrence of *Lactococcus lactis* subsp. *hordniae* has not been reported before in cheeses. It is generally recognized that artisanal cheeses represent one of the ecosystems most used to isolate microorganisms with proper technological potential. These functional characteristics of LAB are strain-dependent [14]. For this reason, genetic identification plays a fundamental role in the selection process of LAB with potential technological properties. Artisanal cheeses derived from raw milk are characterized by different LAB with potential functional properties and certain organoleptic attributes. In recent years, researchers have focused on the isolation of autochthonous LAB from artisanal cheeses made with raw milk without the addition of starter cultures [14]. As in this study, other researchers have isolated strains corresponding to the genera *Lactobacillus* spp., *Lactococcus* spp. and *Leuconostoc* spp. from cheeses made with raw sheep milk [25,26,27].

Different strains of *L. paracasei* have been described before as good candidates for enhancing flavor in cheeses [28,29,30,31,32] and increasing secondary proteolysis and the production of VOCs, such as 2-methylbutanal, diacetyl, 3-methyl-1-butanol, acetic acid and 3-methylbutanoic acid, among others [28]. Probiotic strains of this species (*L. paracasei* KC39) showed an enhanced microstructure (a crumbly structure that increases adhesiveness and springiness) and favorable sensory characteristics of soft cheeses (especially color and general acceptance) [32].

According to Fernández et al. [33] and Cavanagh et al. [34], *L. lactis* subsp. *hordniae* has never been found in dairy products. *L. lactis* subsp. *hordniae* is considerably under-represented in both biological and genomic studies compared to their dairy-associated lactococci counterparts [35]. Usually, only *L. lactis* subsp. *lactis* and *L. lactis* subsp. *cremoris* are considered to be of industrial interest [36]. This makes our identification remarkable. In this work, this microorganism shows its capacity to produce VOCs from other components. In fact, non-dairy *Lactococcus* strains can be observed to produce relatively high abundances of a broad range of important volatile flavor compounds associated with positive attributes in dairy products [37]. This ability to produce a more varied and diverse volatile profile could be beneficial in dairy applications, leading to flavor diversification, generating unique flavor profiles, or indeed to mask off-flavors created by dairy strains [37].

*L. plantarum* was tested to evaluate Manchego cheeses made with different starter cultures, as an adjunct culture to starter culture based on two strains of *L. lactis.* Among the compounds produced are some of those detected in our work, such as benzaldehyde, 1-hexanol, 2-heptanol, 2-pentanone and 2-heptanone. Gómez-Ruiz et al. [38] do not detect, in general, a special contribution of using the adjunct culture of *L. plantarum* together with lactococci. However, more recently, Duan et al. [39] consider *L. plantarum* as an interesting adjunct culture due to its proteolytic activity. Likewise, Jia et al. [40] consider *L. plantarum* among the constituents of a starter with a high incidence in the production of volatiles in goat cheeses.

Leuconostocs were major producers of alcohols and esters. The variations observed between them must be studied for each strain [41]. In fact, *L. mesenteroides* is the microorganism more often isolated from cheeses such as Queijo de Azeitao (Portugal), occurring in 37% of samples of different producers. This prevalence is not surprising since the presence of this species has previously been described in other raw ewe milk and raw cow milk cheeses [42]. Generally, leuconostocs are known to produce aromatic compounds, thus contributing to cheese flavor definition [43].

### 3.2. Identification of VOCs by GC–IMS

VOCs identified in our work are reported in Table 2 and Table 3. Twenty-six compounds were detected in cheese samples (all of them in creamy cheese and twenty-one in matured cheese), fourteen were detected in LAB MRS cultures and twenty-three in skim milk cultures (88.5% of total). Only two compounds (3-methylthiopropanal and 1-butanol) were detected in all the samples analyzed.

**Figure 1:**
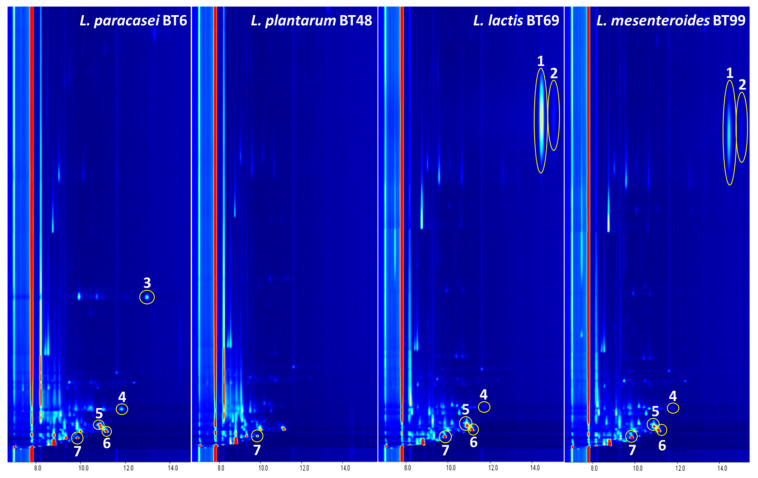
VOCs detected in LAB MRS cultures by GC–IMS. VOCs highlighted: (1) 2-heptanol; (2) 3-hydroxybutan-2-one; (3) 2-heptanone; (4) 3-methyl-1-butanol; (5) 2-butanol; (6) 2-pentanone; (7) 2-butanone.

In LAB MRS cultures, seven of the eleven compounds detected were reported in at least one LAB MRS culture and in the MRS control medium. However, the other seven compounds were detected exclusively in the samples from at least one LAB MRS culture (Figure 1). These data indicate that at least these compounds (2-heptanone, 2-pentanone, 2-butanone, 3-hydroxybutan-2-one, 2-butanol, 2-heptanol and 3-methyl-1-butanol) could be generated by LAB activity.

**Figure 2:**
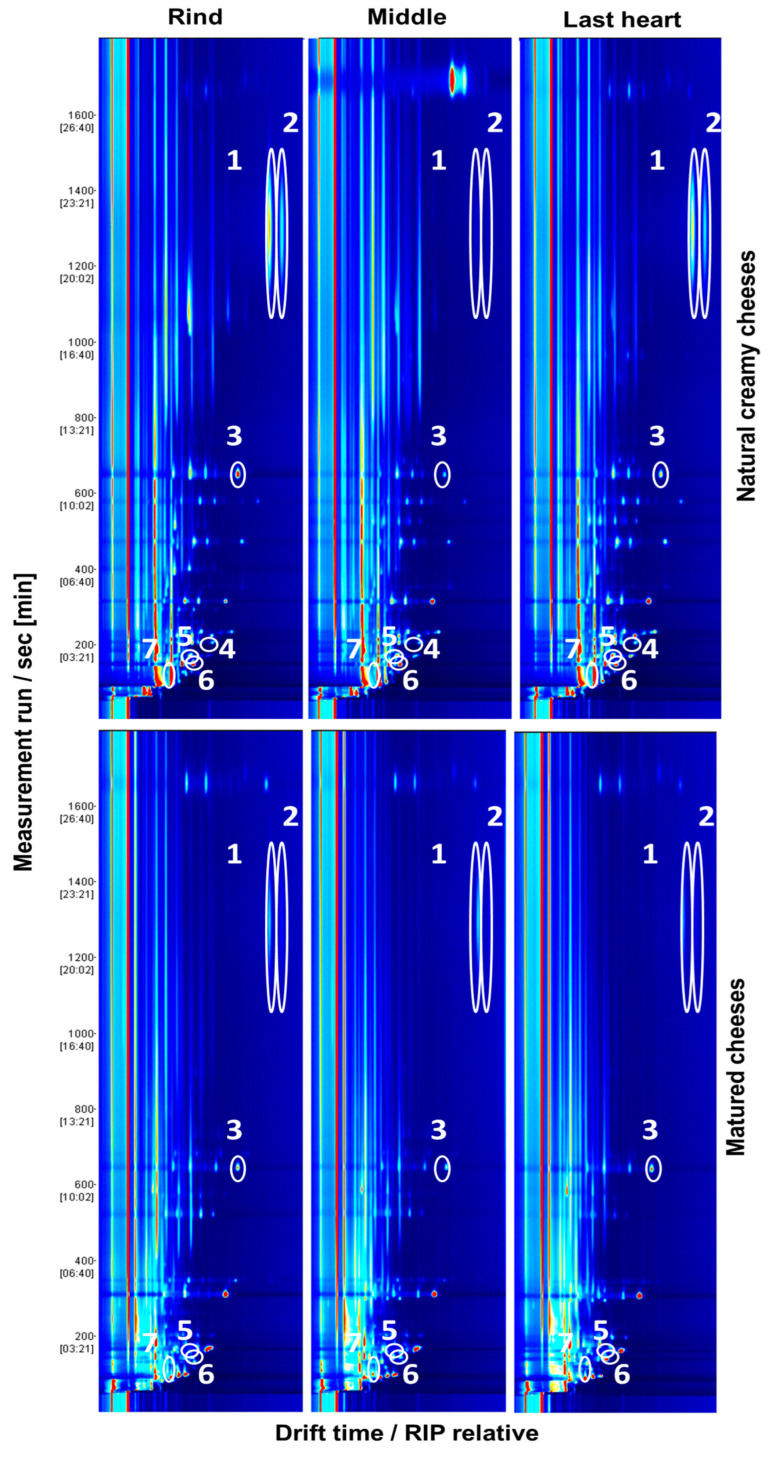
VOCs detected in artisanal raw sheep milk (natural creamy or matured) cheeses by GC–IMS. VOCs highlighted: (1) 2-heptanol; (2) 3-hydroxybutan-2-one; (3) 2-heptanone; (4) 3-methyl-1-butanol; (5) 2-butanol; (6) 2-pentanone; (7) 2-butanone.

**Figure 3:**
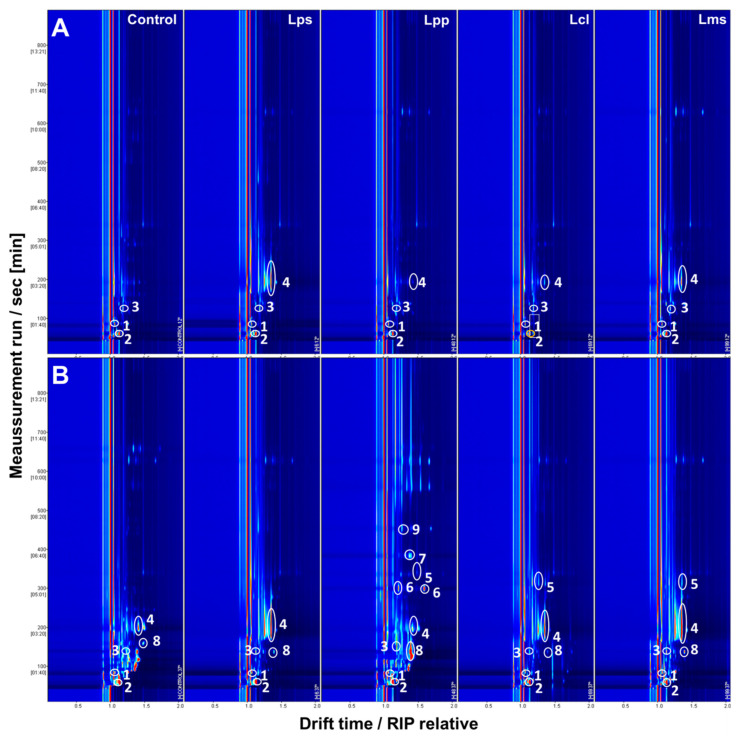
VOCs detected in skim milk LAB cultures after incubation 48 h at 12 °C and 37 °C by GC–IMS. (**A**) At 12 °C; (**B**) At 37 °C; VOCs highlighted: (1) 2-methylbutanal; (2) 2-methylpropanal; (3) Acetaldehyde; (4) Pentan-1-ol; (5) Hexanal; (6) 2-hexanone; (7) Ethyl butanoate; (8) Ethyl propanoate; (9) Propyl butanoate.

The seven compounds detected exclusively in MRS cultures of LAB were recorded in at least one cheese sample (Figure 1, Figure 2 and Figure 3). These compounds were investigated to determine whether they occur in different types of artisanal cheeses (creamy and matured). The presence of these compounds was also evaluated in different parts of the cheese (rind, middle (inner) and last heart (center) of the sample). Figure 2 shows the topographical maps obtained for the analysis of a natural creamy cheese and a matured cheese in their three parts. Six of the seven compounds previously identified in the LAB MRS cultures were found in these cheese samples. As can be seen in Figure 2, 3-methyl-1-butanol was not detected in matured cheese samples.

With respect to the bacterial cultures in skim milk, ten of the twenty-three VOCs detected were identified in both the skim milk control and at least one skim milk culture. The other thirteen compounds were detected exclusively in the samples from at least one LAB skim milk culture (Table 2 and Table 3). When the effect of bacterial metabolism of milk at different temperatures (12 and 37 °C) was studied, it was observed that at least these compounds (2-hexanone, esters except ethyl acetate, 2-methylbutanal (at 12 °C), hexanal, 2-methylpropanal, acetaldehyde, 2-butanol, 2-heptanol, n-hexanol (at 37 °C) and pentan-1-ol) were identified by GC–IMS, except for 2-butanol and 2-heptanol (also identified in MRS cultures and appeared to be related to the LAB strain). All compounds detected exclusively in LAB skim milk cultures were found in at least one cheese sample (Figure 2 and Figure 3). The importance of the milk source for LAB activity is demonstrated by the detection of eleven VOCs in skim milk cultures undetected in LAB MRS cultures: ethyl hexanoate, ethyl propanoate and propyl butanoate (only in *L. plantarum* at 37 °C), 2-methylbutanal, butanal, hexanal (only in *L. lactis* subsp. *hordniae*), 2-methylpropanal, acetaldehyde, 2-methyl-1-propanol, pentan-1-ol and acetic acid (the only organic acid we have checked).

Considering VOCs detected in creamy cheeses, twenty-three VOCs (88.5% of total) were detected in skim milk cultures (100% of VOCs detected in this kind of cheese). *L. plantarum* is the most important producer of esters in these last cases. This activity is clearly differentiated from that attributed to Leuconostoc. Furthermore, there were four VOC compounds detected in cheeses only detected in skim milk cultures of *L. plantarum*: 2-hexanone, ethyl butanoate, ethyl hexanoate and propyl butanoate. The activity of both microorganisms in the production of esters and aldehydes from a dairy source (MRS culture) was not detected, except for the cases where even control samples include these compounds (only ethyl butanoate, benzaldehyde and 3-methylthiopropanal). Results of both microorganisms could be statistically differentiated (*p* < 0.05). VOC production of both microorganisms agrees completely with the detection of these compounds in creamy cheese.

Regarding matured cheese samples, nineteen VOCs (73% of total) were detected in skim milk cultures. Results are not correlated for 2-hexanone, ethyl butanoate, ethyl hexanoate, benzaldehyde, 3-methyl-1-butanol and acetic acid. These differences could be related to time required to maturation as well as interactions during this process. All the compounds detected by the activity of both microorganisms are present in the matured cheese, with only two exceptions: ethyl propanoate and 3-methyl-1-butanol. A total of 80% of the VOCs detected in this cheese are found from *L. paracasei* and 90% from *L. lactis* subsp. *Hordniae*. Differences between both LAB profiles are 22%. Differences between behavior of LAB at 12 °C or 37 °C are found, but we cannot demonstrate statistical differences.

### 3.3. Discussion Based on Groups of VOCs

In general, in our work, the production of ketones could be not necessarily dependent on LAB activities because of their detection in skim milk control samples. In fact, ketones are usual contributors to the typical aroma of dairy products [21], although it is generally difficult to determine their origin in cheeses. Some of these compounds appear to derive from animal feed (by cutting the grass or upon drying) or proteolysis (due, for instance, to coagulants used) [5], while others such as methyl ketones seem to be formed during cheese ripening from the metabolic activity of the dominant microbiota [22]. In fact, the detection of four ketones in MRS cultures without occurring in control samples shows the potential activity of all the LAB studied in this sense. Fruity and floral notes are associated with ketones, so the presence of these volatile compounds can be considered a positive for cheese flavor. 2-Butanone, with a butterscotch odor, was identified as the main odorant in cheddar cheese and 2-heptanone, with an herbaceous odor, is an important flavor compound of Emmental and natural and creamy Gorgonzola cheeses [24]. All VOCs corresponding to this group detected in our cheeses, such as 2-butanone, 2-pentanone, 2-hexanone, 2-heptanone and 3-hydroxybutan-2-one, were also found in the MRS LAB cultures and in the skim milk cultures (Table 2 and Table 3). 2-Pentanone was previously described as a secondary metabolite by LAB [20].

One of the main classes of volatile components in cheeses are esters [5]. Esters in fermented dairy products are formed between short-to-medium-chain fatty acids and available primary and secondary alcohols derived from lactose fermentation or from amino acid catabolism [16]. These reactions are performed either by microorganisms or by chemical reactions [44]. The non-detection of esters from the MRS LAB cultures studied in our work is consistent with the absence of lactose in MRS broth. In our work, and as we have described, *L. plantarum* is the most important producer of esters in creamy cheese samples. None of the skim milk cultures at 12 °C produced esters. However, at 37 °C, *L. plantarum* (with the exception of ethyl acetate) and the rest of LAB studied (in the case of ethyl propanoate) show activity, which is also expressed in cheeses, especially creamy cheese. The occurrence of ethyl acetate in these creamy cheeses could be attributed to chemical reactions [44]. No derived secondary alcohols are present. On the other hand, their occurrence in the cheeses studied is expected. In fact, ethyl propanoate and ethyl butanoate predominated in natural creamy cheese and ethyl butanoate and ethyl hexanoate were predominant in both types of cheeses analyzed. Esters, which are produced by esterification of an acid and an alcohol, were the most identified group in cheeses, mainly in creamy natural cheese. Researchers considered them one of the main groups of compounds isolated from the headspace of “Torta del Casar” cheeses [21]. According to these authors, some LAB are involved in this production, as it is our case. These compounds confer, in a synergistic way, floral and fruity notes that probably contribute to the balance of cheese flavor by minimizing the sharpness imparted by the acids [22].

Aldehydes present transitory nature and do not accumulate in cheese since they tend to reduce into the corresponding alcohols or, alternatively, oxidize into the respective acids [45]. However, a diversity of aldehydes in cheese samples was found. In natural creamy cheese, hexanal and 2-methylpropanal were detected. In both matured and creamy cheeses, we detected 2-methylbutanal, butanal, octanal and acetaldehyde (Table 2 and Table 3). These compounds are main products of the autoxidation of unsaturated fatty acids, playing a key role in the development of cheese flavor. For instance, octanal could be produced by the autooxidation of oleic acid, and it is characterized by a green grass-like and herbaceous aroma [11]. On the other hand, 2-methylbutanal, with its typically aldehydic odor, may derive from the catabolism of amino acids such as isoleucine [21]. In our study, the absence of fatty acids in MRS broth prevents the occurrence of aldehydes in MRS LAB cultures separately, excluding benzaldehyde and 3-methylthiopropanal, whose fatty acids are probably produced from peptone components. In skim milk cultures, aldehydes are produced in most of the samples, except for benzaldehyde and octanal. The activity of LAB studied in milk produce acetaldehyde, an important organoleptic compound.

In general, raw milk cheeses contain greater amounts of alcohols than pasteurized milk cheeses and this is due to their higher microbial diversity [17]. The formation of primary and secondary alcohols in cheeses could be due to lactose fermentation or generated by dehydrogenation of aldehydes and ketones due to the strong reducing conditions of cheese matrices [18]. Alcohols can produce acids, keto acids and carbonyls by catabolism [22]. In our study, n-hexanol, pentan-1-ol and 3-methyl-1-butanol were detected in natural creamy cheeses and 2-butanol, 2-methyl-1-propanol, 2-heptanol and 1-butanol were detected in both cheeses (Table 2 and Table 3). Specifically, various alcohols were detected in MRS LAB and skim milk cultures, as well as in cheese samples. Secondary alcohols such as 2-heptanol and 2-butanol were detected from *L. lactis* subsp. *Hordniae*, *L. mesenteroides* and both cheeses, and from *L. paracasei*, *L. lactis* subsp. *Hordniae*, *L. mesenteroides* and both cheeses, respectively. 2-Heptanol has been identified as a key odorant of Gorgonzola and Grana Padano cheeses and it was the alcohol detected in the highest concentrations in semi-hard Spanish goat cheeses. 2-Butanol was the highest secondary alcohol isolated in the artisanal Manchego cheese [24]. In our study, 3-methyl-1-butanol was detected from *L. paracasei*, *L. lactis* subsp. *Hordniae*, *L. mesenteroides* (MRS cultures), in all the skim milk cultures and in creamy cheese. The presence of branched-chain primary alcohols, such as 3-methyl-1-butanol, indicates the reduction in the aldehyde produced from leucine. This compound has been detected in raw goat’s milk cheeses and confers a pleasant fresh cheese flavor [24]. According to Gallegos et al. [11], 1-hexanol, a primary alcohol, is mainly produced by the reduction in its corresponding aldehyde and methyl ketone. In all cheeses, these alcohols give a particular flavor, which makes them distinctive and recognizable by consumers.

Generally, in cheeses, carboxylic acids are abundant volatile compounds. They give acidic, fatty, pungent, balsamic and vinegary odors [46]. Linear carboxylic acids, such as acetic, butanoic, hexanoic and octanoic, decanoic, butyric and acetic acids have often been detected in cheeses. However, the method used in our study has limited the detection of these types of compounds. Acetic acid was the only compound studied that could be identified in the samples of creamy natural cheese, as well as the skim milk cultures (Table 2 and Table 3). This is not strange, because this compound derives mainly from the early fermentation of lactate [47].

## 4. Conclusions

Predicting the ability of different characterized LAB to produce aromas and the identification of the VOCs responsible for flavor in cheeses are important aspects to consider when selecting strains with optimal aromatic properties, resulting in the diversification of cheese products. In our work, the contribution of each LAB studied to the aroma profile of the cheeses can be described according to their different VOC profiles. Differences between LAB behavior in each cheese are shown, especially between LAB involved in creamy cheeses. Only *L. lactis* subsp. *Hordniae* and *L. mesenteroides* show the same VOC profile in MRS cultures, but they participate in different cheeses, and also show two differences in VOC production in skim milk cultures (especially pentan-1-ol).

The combination of Sanger sequencing in identifying LAB from raw milk cheeses with GC–IMS to determinate the VOCs produced helped us to describe cheeses and to differentiate the potential role of each microorganism in their volatilome. Further studies will be useful to determinate the production of other VOCs, especially in searching for different acids. Finally, as far as we know, the occurrence of *Lactococcus lactis* subsp. *Hordniae* in cheese is reported for first time.

## Figures and Tables

**Table 1:** Reference compounds of selected cheeses for analysis of LAB samples and raw sheep milk cheese.

Chemical Group	VOC	RI	t_R_	t_D_	t_D_ (Database)	Reference *
Ketones	2-Heptanone	892.2	647.6; 647.0	1.3; 1.6	1.3; 1.6	[16,17,18,19]
	2-Hexanone	784.2	270.0; 292.0	1.2; 1.5	1.2; 1.5	[16,18]
	2-Pentanone	688.6	140.5; 148.7	1.1; 1.4	1.1; 1.4	[16,18,20]
	2-Butanone	589.4	96.2; 89.3	1.1; 1.3	1.1; 1.2	[7,17,20,21,22]
	3-Hydroxybutan-2-one	715.12	1384.2	1.8	NA	[7,23]
Esters	Ethyl butanoate	794.9	302.3; 300.6	1.2; 1.6	1.2; 1.6	[16,17,18,20,22,23]
	Ethyl hexanoate	999.9	1598.8; 1603.4	1.3; 1.8	1.3; 1.8	[7,16,17,18,22,23]
	Ethyl propanoate	707.7	161.0; 162.6	1.2; 1.5	1.1; 1.4	[16]
	Propyl butanoate	895.9	660.1; 663.7	1.3; 1.7	NA	[18,22]
	Ethyl acetate	668.8; 673.2	138.1; 140.6	1.2; 1.4	1.1; 1.3	[16,17,18,20]
Aldehydes	2-Methylbutanal	694.6	119.5; 120.9	1.2; 1.4	NA	[17,18,19,24]
	Benzaldehyde	960.9	1083.9; 1087.7	1.1; 1.5	1.1; 1.5	[7,16,17,19,20,22,23]
	Butanal	602.6; 602.6	85.5; 85.1	1.1; 1.3	1.1; 1.3	[17,20]
	Hexanal	800.2; 800.2	288.9; 291.5	1.3; 1.6	1.2; 1.6	[17,18,19]
	2-Methylpropanal	551.8; 551.8	75.9; 75.9	1.1; 1.3	1.1; 1.3	[18,20]
	3-Methylthiopropanal	907.7; 907.7	719.0; 721.9	1.1; 1.4	1.1; 1.4	[19]
	Octanal	128.2	1001.9; 1001.9	1.4; 1.8	1.4; 1.8	[4,18,23]
	Acetaldehyde	784.7; 785.5	293.4; 296.1	1.2; 1.4	NA	[18,20,23]
Alcohols	2-Butanol	602.7; 602.7	131.7; 133.0	1.2; 1.4	NA	[16,17,18,22]
	2-Methyl-1-propanol	629.0; 629.0	102.9; 104.4	1.2; 1.4	NA	[18,20,22,23]
	2-Heptanol	970.5; 970.5	1329.0; 1335.3	1.4; 1.8	NA	[16,17,18,22,23]
	n-Hexanol	867.4; 867.4	561.8; 566.3	1.3; 1.6	1.9; 1.6	[7,16,17,18,20,23]
	n-Butanol	660.4; 660.4	129.2; 125.5	1.2; 1.4	1.2; 1.4	[16,17,21,22]
	1-Pentanol	765.9; 765.9	250.9; 252.5	1.2; 1.5	1.2; 1.5	[17,23]
	3-Methyl-1-butanol	724.0; 723.7	133.8; 135.2	1.2; 1.4	NA	[16,17,18,19,21,22]
Acids	Acetic acid	621.3684	93.05; -	1.0; -	1.0; 1.5	[16,19,20,21,23]

RI, retention Index; t_R_ (s), retention time in seconds; t_D_ (ms), drift time in milliseconds; t_D_ (database), drift time of database in milliseconds; NA, data not available in database. * See References section.

**Table 2:** VOCs detected in LAB cultures and creamy raw sheep milk cheese.

Chemical Group	VOC	MRS Media			Milk at 37 °C			Milk at 12 °C			Creamy Cheese		
		CT	Lpp	Lms	CT	Lpp	Lms	CT	Lpp	Lms	Rind	Inner	Center
Ketones	2-Heptanone	-	-	-	+	+	+	+	+	+	+	+	+
	2-Hexanone	+	+	+	-	+	-	-	-	-	+	+	+
	2-Pentanone	-	-	+	+	+	+	+	+	+	+	+	+
	2-Butanone	-	+	+	+	+	+	+	+	+	+	+	+
	3-Hydroxybutan-2-one	-	-	+	+	+	+	+	+	+	+	+	+
Esters	Ethyl butanoate	+	+	+	-	+	-	-	-	-	+	+	+
	Ethyl hexanoate	-	-	-	-	+	-	-	-	-	+	+	+
	Ethyl propanoate	-	-	-	-	+	+	-	-	-	+	+	+
	Propyl butanoate	-	-	-	-	+	-	-	-	-	+	+	+
	Ethyl acetate	-	-	-	-	-	-	-	-	-	+	+	+
Aldehydes	2-Methylbutanal	-	-	-	-	+	+	-	+	+	+	+	+
	Benzaldehyde	+	+	+	-	-	-	-	-	-	+	+	+
	Butanal	-	-	-	+	+	-	-	+	-	-	+	+
	Hexanal	-	-	-	-	-	-	-	-	+	+	+	+
	2-Methylpropanal	-	-	-	-	+	+	-	+	+	+	+	+
	3-Methylthiopropanal	+	+	+	+	+	+	+	+	+	+	+	+
	Octanal	-	-	-	-	-	-	-	-	-	-	-	+
	Acetaldehyde	-	-	-	-	+	+	-	+	+	+	+	+
Alcohols	2-Butanol	-	-	+	-	-	+	-	-	+	+	+	+
	2-Methyl-1-propanol	-	-	-	+	+	+	+	+	+	+	+	+
	2-Heptanol	-	-	+	-	-	+	-	-	+	+	+	+
	n-Hexanol	+	+	+	-	+	-	+	+	+	+	+	+
	1-Butanol	+	+	+	+	+	+	+	+	+	+	+	+
	Pentan-1-ol	-	-	-	-	+	+	-	+	-	+	+	+
	3-Methyl-1-butanol	-	-	+	+	+	+	+	+	+	+	+	+
Acids	Acetic acid	-	-	-	+	+	+	+	+	+	+	+	+

Lpp: Lactiplantibacillus plantarum; Lms: Leuconostoc mesenteroides; + VOC detected; - VOC not detected.

**Table 3:** VOCs detected in LAB cultures and raw sheep milk matured cheese.

Chemical Group	VOC	MRS Media			Milk at 37 °C			Milk at 12 °C			Matured Cheese		
		CT	Lps	Lcl	CT	Lps	Lcl	CT	Lps	Lcl	Rind	Inner	Center
Ketones	2-Heptanone	-	+	-	+	+	+	+	+	+	+	+	+
	2-Hexanone	+	+	+	-	-	-	-	-	-	+	+	+
	2-Pentanone	-	+	+	+	+	+	+	+	+	+	+	-
	2-Butanone	-	+	+	+	+	+	+	+	+	+	+	+
	3-Hydroxybutan-2-one	-	-	+	+	+	+	+	+	+	+	+	+
Esters	Ethyl butanoate	+	+	+	-	-	-	-	-	-	+	+	+
	Ethyl hexanoate	-	-	-	-	-	-	-	-	-	+	+	+
	Ethyl propanoate	-	-	-	-	+	+	-	-	-	-	-	-
	Propyl butanoate	-	-	-	-	-	-	-	-	-	-	-	-
	Ethyl acetate	-	-	-	-	-	-	-	-	-	-	-	-
Aldehydes	2-Methylbutanal	-	-	-	-	+	+	-	+	+	+	+	+
	Benzaldehyde	+	+	+	-	-	-	-	-	-	+	+	+
	Butanal	-	-	-	+	+	-	-	+	+	+	+	+
	Hexanal	-	-	-	-	-	+	-	-	-	+	+	+
	2-Methylpropanal	-	-	-	-	+	+	-	+	+	+	+	+
	3-Methylthiopropanal	+	+	+	+	+	+	+	+	+	+	+	+
	Octanal	-	-	-	-	-	-	-	-	-	+	+	+
	Acetaldehyde	-	-	-	-	+	+	-	+	+	+	+	+
Alcohols	2-Butanol	-	+	+	-	+	+	-	+	+	+	+	+
	2-Methyl-1-propanol	-	-	-	+	+	+	+	+	+	+	+	+
	2-Heptanol	-	-	+	-	-	+	-	-	+	+	+	+
	n-Hexanol	+	-	+	-	+	-	+	+	+	+	+	+
	1-Butanol	+	+	+	+	+	+	+	+	+	+	+	+
	Pentan-1-ol	-	-	-	-	+	-	-	-	+	-	-	-
	3-Methyl-1-butanol	-	+	+	+	+	+	+	+	+	-	-	-
Acids	Acetic acid	-	-	-	+	+	+	+	+	+	-	-	-

Lps: Lacticaseibacillus paracasei; Lcl: Lactococcus lactis subsp. hordniae; + VOC detected; - VOC not detected.

## Data Availability

Data is contained within the article.

## References

[1] Colonna A., Durham C., Meunier-Goddik L. (2011). Factors affecting consumers’ preferences for and purchasing decisions regarding pasteurized and raw milk specialty cheeses. J. Dairy Sci..

[2] AMRC (Ag Marketing Resource Center) (2022). Cheese Industry Profile. https://www.agmrc.org/commodities-products/livestock/dairy/cheese-industry-profile.

[3] Mohapatra A., Shinde A.K., Singh R. (2019). Sheep milk: A pertinent functional food. Small Rumin. Res..

[4] Gallegos J., Arce C., Jordano R., Arce L., Medina L.M. (2017). Target identification of volatile metabolites to allow the differentiation of lactic acid bacteria by gas chromatography-ion mobility spectrometry. Food Chem..

[5] Soltani M., Sahingil D., Gokce Y., Hayaloglu A.A. (2016). Changes in volatile composition and sensory properties of Iranian ultrafiltered white cheese as affected by blends of *Rhizomucor miehei* protease or camel chymosin. J. Dairy Sci..

[6] Zheng J., Wittouck S., Salvetti E., Franz C.M., Harris H.M., Mattarelli P., O’Toole P.W., Pot B., Vandamme P., Walter J. (2020). A taxonomic note on the genus *Lactobacillus*: Description of 23 novel genera, emended description of the genus *Lactobacillus* Beijerinck 1901, and union of Lactobacillaceae and Leuconostocaceae. Int. J. Syst. Evol. Microbiol..

[7] Gaglio R., Franciosi E., Todaro A., Guarcello R., Alfeo V., Randazzo C.L., Settanni L., Todaro M. (2020). Addition of selected starter/non-starter lactic acid bacterial inoculums to stabilise PDO Pecorino Siciliano cheese production. Food Res. Int..

[8] Galimberti A., Casiraghi M., Bruni I., Guzzetti L., Cortis P., Berterame N.M., Labra M. (2019). From DNA barcoding to personalized nutrition: The evolution of food traceability. Curr. Res. Food Sci..

[9] Contreras M.M., Jurado-Campos N., Arce L., Arroyo-Manzanares N. (2019). A robustness study of calibration models for olive oil classification: Targeted and non-targeted fingerprint approaches based on GC-IMS. Food Chem..

[10] Arce L., Gallegos J., Garrido-Delgado R., Medina L.M., Sielemann S., Wortelmann T. (2014). Ion mobility spectrometry a versatile analytical tool for metabolomics applications in food science. Curr. Metab..

[11] Gallegos J., Garrido-Delgado R., Arce L., Medina L.M. (2015). Volatile metabolites of goat cheeses determined by Ion Mobility Spectrometry. Potential applications in quality control. Food Anal. Methods.

[12] Bertuzzi A.S., McSweeney P.L.H., Rea M.C., Kilcawley K.N. (2018). Detection of volatile compounds of cheese and their contribution to the flavor profile of surface-ripened cheese. Compr Rev. Food Sci. Food Saf..

[13] Kilcawley K.N., Faulkner H., Clarke H.J., O’Sullivan M.G., Kerry J.P. (2018). Factors influencing the flavour of bovine milk and cheese from grass based versus non-grass based milk production systems. Foods.

[14] Ruiz-Moyano S., Goncalves dos Santos M.T., Galván A.I., Merchán A.V., González E., Córdoba M.G., Benito M.J. (2019). Screening of autochthonous lactic acid bacteria strains from artisanal soft cheese: Probiotic characteristics and prebiotic metabolism. LWT..

[15] Beck T.F., Mullikin J.C., Biesecker L.G., Progra N.C.S. (2016). Systematic evaluation of Sanger validation of next-generation sequencing variants. Clin. Chem..

[16] Cozzolino R., Martignetti A., De Giulio B., Malorni L., Addeo F., Picariello G. (2021). SPME GC-MS monitoring of volatile organic compounds to assess typicity of Pecorino di Carmasciano ewe-milk cheese. Int. J. Dairy Technol..

[17] Guarrasi V., Sannino C., Moschetti M., Bonanno A., Di Grigoli A., Settanni L. (2017). The individual contribution of starter and non-starter lactic acid bacteria to the volatile organic compound composition of Caciocavallo Palermitano cheese. Int. J. Food Microbiol..

[18] Marilley L., Casey M.G. (2004). Flavours of cheese products: Metabolic pathways, analytical tools and identification of producing strains. Int. J. Food Microbiol..

[19] Luz C., D’Opazo V., Quiles J.M., Romano R., Mañes J., Meca G. (2020). Biopreservation of tomatoes using fermented media by lactic acid bacteria. LWT..

[20] Štefániková J., Árvay J., Miškeje M., Kačániová M. (2020). Determination of volatile organic compounds in Slovak Brynda cheese by the electronic nose and the headspace solid-phase microextraction gas chromatography-mass spectrometry. Slovak J. Food Sci..

[21] Delgado-Martínez F., Carrapiso A.I., Contador R., Rosario Ramírez M. (2019). Volatile compounds and sensory changes after high pressure processing of mature “Torta del Casar” (raw ewe’s milk cheese) during refrigerated storage. Innov. Food Sci. Emerg. Technol..

[22] Bozoudi D., Kondyli E., Claps S., Hatzikamari M., Michaelidou A., Biliaderis C.G., Litopoulou-Tzanetaki E. (2018). Compositional characteristics and volatile organic compounds of traditional PDO Feta cheese made in two different mountainous areas of Greece. Int. J. Dairy Technol..

[23] Reale A., Di Renzo T., Boscaino F., Nazzaro F., Fratianni F., Aponte M. (2019). Lactic acid bacteria biota and aroma profile of Italian traditional sourdoughs from the irpinian area in Italy. Front. Microbiol..

[24] Delgado F., Rodríguez-Pinilla J., Márquez G., Roa I., Ramírez R. (2015). Physicochemical, proteolysis and texture changes during the storage of a mature soft cheese treated by high-pressure hydrostatic. Eur. Food Res. Technol..

[25] Kirmaci H.A., Ozer B.H., Akcelik M., Akcelik N. (2016). Identification and characterisation of lactic acid bacteria isolated from traditional Urfa cheese. Int. J. Dairy Technol..

[26] Ramírez-López C., Vélez-Ruiz J.F. (2016). Isolation, characterization and selection of autochthonous lactic acid bacteria from goat milk and fresh artisanal-goat cheese. Inf. Tecnol..

[27] Zoumpopoulou G., Papadimitriou K., Alexandraki V., Mavrogonatou E., Alexopoulou K., Anastasiou R., Georgalaki M., Kletsas D., Tsakalidou E., Giaouris E. (2020). The microbiota of Kalathaki and Melichloro Greek artisanal cheeses comprises functional lactic acid bacteria. LWT.

[28] Peralta G.H., Wolf I.V., Bergamini C.V., Perotti M.C., Hynes E.R. (2014). Evaluation of volatile compounds produced by *Lactobacillus paracasei* I90 in a hard-cooked cheese model using solid-phase microextraction. Dairy Sci. Technol..

[29] Poveda J.M., Nieto-Arribas P., Seseña S., Chicón R., Castro L., Palop L., Cabezas L. (2014). Volatile composition and improvement of the aroma of industrial Manchego cheese by using *Lactobacillus paracasei* subsp. *paracasei* as adjunct and other autochthonous strains as starters. Eur. Food Res. Technol..

[30] Stefanovic E., Kilcawley K.N., Roces C., Rea M.C., O’Sullivan M., Sheehan J.J., McAuliffe O. (2018). Evaluation of the potential of *Lactobacillus paracasei* adjuncts for flavor compounds development and diversification in short-aged Cheddar cheese. Front. Microbiol..

[31] Terpou A., Bosnea L., Kanellaki M., Plessas S., Bekatorou A., Bezirtzoglou E., Koutinas S.A. (2018). Growth capacity of a novel potential probiotic *Lactobacillus paracasei* K5 strain incorporated in industrial white brined cheese as an adjunct culture. J. Food Sci..

[32] Shehata M.G., Abd El-Aziz N.M., Darwish A.G., El-Sohaimy S.A. (2022). *Lacticaseibacillus paracasei* KC39 immobilized on prebiotic wheat bran to manufacture functional soft white cheese. Fermentation.

[33] Fernández E., Alegría A., Delgado S., Martín M.C., Mayo B. (2011). Comparative phenotypic and molecular genetic profiling of wild *Lactococcus lactis* subsp. *lactis* strains of the *L. lactis* subsp. *lactis* and *L. lactis* subsp. *cremoris* genotypes, isolated from starter-free cheeses made of raw milk. Appl. Environ. Microbiol..

[34] Cavanagh D., Casey A., Altermann E., Cotter P.D., Fitzgerald G.F., McAuliffe O. (2015). Evaluation of *Lactococcus lactis* Isolates from Nondairy Sources with Potential Dairy Applications Reveals Extensive Phenotype-Genotype Disparity and Implications for a Revised Species. Appl. Environ. Microbiol..

[35] Kelleher P., Bottacini F., Mahony J., Kilcawley K.N., Van Sinderen D. (2017). Comparative and functional genomics of the *Lactococcus lactis* taxon; insights into evolution and niche adaptation. BMC Genom..

[36] Laroute V., Tormo H., Couderc C., Mercier-Bonin M., Le Bourgeois P., Cocaign-Bousquet M., Daveran-Mingot M.L. (2017). From genome to phenotype: An integrative approach to evaluate the biodiversity of *Lactococcus lactis*. Microorganisms.

[37] McAuliffe O. (2018). Symposium review: Lactococcus lactis from non dairy sources: Their genetic and metabolic diversity and potential applications in cheese. J. Dairy Sci..

[38] Gómez-Ruiz J.A., Cabezas L., Martínez-Castro I., González-Viñas M.A., Poveda J.M. (2008). Influence of a defined-strain starter and *Lactobacillus plantarum* as adjunct culture on volatile compounds and sensory characteristics of Manchego cheese. Eur. Food Res. Technol..

[39] Duan C., Li S., Zhao Z., Wang C., Zhao Y., Yang G., Niu C., Gao L., Liu X., Zhao L. (2019). Proteolytic activity of *Lactobacillus plantarum* strains in Cheddar cheese as adjunct cultures. J. Food Prot..

[40] Jia R., Zhang F., Song Y., Lou Y., Zhao A., Liu Y., Peng H., Hui Y., Ren R., Wang B. (2021). Physicochemical and textural characteristics and volatile compounds of semihard goat cheese as affected by starter cultures. J. Dairy Sci..

[41] Pogačić T., Maillard M.B., Leclerc A., Hervé C., Chuat V., Valence F., Thierry A. (2016). *Lactobacillus* and *Leuconostoc* volatilomes in cheese conditions. Appl. Microbiol. Biotechnol..

[42] Cardinali F., Ferrocino I., Milanović V., Belleggia L., Rita Corvaglia M., Garofalo C., Foligni R., Mannozzi C., Mozzon M., Cocolin L. (2021). Microbial communities and volatile profile of Queijo de Azeitão PDO cheese, a typical Mediterranean thistle curdled cheese from Portugal. Food Res. Int..

[43] Van Mastrigt O., Egas R.A., Abee T., Smid E.J. (2019). Aroma formation in retentostat co-cultures of *Lactococcus lactis* and *Leuconostoc mesenteroides*. Food Microbiol..

[44] Majcher M.A., Goderska K., Pikul J., Jelen H.H. (2011). Changes in volatile, sensory and microbial profiles during preparation of smoked ewe cheese. J. Sci. Food Agric..

[45] Coda R., Brechany E., De Angelis M., De Candia S., Di Cagno R., Gobbetti M. (2006). Comparison of the compositional, microbiological, biochemical, and volatile profile characteristics of nine Italian ewes’ milk cheeses. J. Dairy Sci..

[46] The Good Scents Company Information System Providing Information for the Flavor, Fragrance, Food and Cosmetic Industries. http://www.thegoodscentscompany.com/index.html.

[47] Curioni P.M., Bosset J.O. (2002). Key odorants in various cheese types as determined by gas chromatography-olfactometry. Int. Dairy J..

